# A Perspective on Innovating the Chemistry Lab Bench

**DOI:** 10.3389/frobt.2020.00024

**Published:** 2020-02-25

**Authors:** Alexander G. Godfrey, Samuel G. Michael, Gurusingham Sitta Sittampalam, Gergely Zahoránszky-Köhalmi

**Affiliations:** National Center for Advancing Translational Sciences, National Institutes of Health, Bethesda, MD, United States

**Keywords:** automated chemical synthesis, retrosynthetic analysis, reaction planning, reagent delivery, bench chemistry

## Abstract

Innovating on the design and function of the chemical bench remains a quintessential challenge of the ages. It requires a deep understanding of the important role chemistry plays in scientific discovery as well a first principles approach to addressing the gaps in how work gets done at the bench. This perspective examines how one might explore designing and creating a sustainable new standard for advancing automated chemistry bench itself. We propose how this might be done by leveraging recent advances in laboratory automation whereby integrating the latest synthetic, analytical and information technologies, and AI/ML algorithms within a standardized framework, maximizes the value of the data generated and the broader utility of such systems. Although the context of this perspective focuses on the design of advancing molecule of potential therapeutic value, it would not be a stretch to contemplate how such systems could be applied to other applied disciplines like advanced materials, foodstuffs, or agricultural product development.

## Introduction

**Figure 1:**
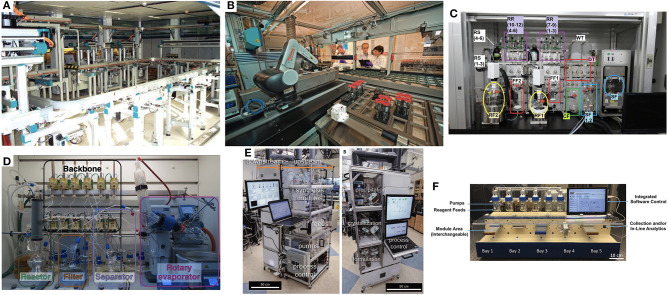
**(A)** SynCAR (Weber et al., 2005), **(B)** ASL (Godfrey et al., 2013), **(C)** ChemKonzert (Masui et al., 2017), and most recently the **(D)** ChemPuter (Steiner et al., 2019) **(E)** MIT's Pharmacy on Demand module (Adamo et al., 2016), **(F)** MIT's Reconfigurable Self-Optimizing Reactor (Bedard et al., 2018) (All images used with permission from the respective publishers).

The central thesis posited by this perspective is the following question: What would it take to make the traditional chemistry hood no longer the central element from which chemistry discovery and critical productivity emanates? In other words, what would it take to build a platform that chemists would seek out in their day-to-day work in preference to the traditional chemistry fume hood? Notwithstanding, there are many aspects of work of the synthetic chemist that have enjoyed significant infusions of technological advancements in the form of specialized chemical synthesis platforms designed and standardized around very specific transformations [peptides (Merrifield, 1985), MIDA-boronate couplings (Li et al., 2015), oligosaccharides (Plante et al., 2001)] to more generalized platforms [Figure 1A SynCAR (Weber et al., 2005), Figure 1B ASL (Godfrey et al., 2013), Figure 1C ChemKonzert (Masui et al., 2017), and most recently the Figure 1D ChemPuter (Steiner et al., 2019)]. The ever increasing interest and value of flow chemistry technologies (Bogdan and Dombrowski, 2019) has led to highly integrated systems as well [Figure 1E MIT's Pharmacy on Demand module (Adamo et al., 2016), Figure 1F MIT's Reconfigurable Self-Optimizing Reactor (Bedard et al., 2018)]. High throughput reaction screening platforms have recently seen a resurgence of interest (Buitrago Santanilla et al., 2015; Perera et al., 2018; Isbrandt et al., 2019). But it is interesting to note how often one can find footnotes to highly automated and productive platforms being fed by substrates that have been synthesized manually. This is not directed as a critique and the incredible value provided by such platforms, but to the central question of what it would take to alleviate this manual synthesis support element still so pervasive in modern day labs.

The answer may in part come in the form of more highly integrated modular robotic platforms designed to execute both batch- and flow-mediated chemistries.

The discovery and development of chemistry methodologies remains critical to the advancement of automated synthesis. Automated solid-phase peptide synthesis, despite having initially developed well over 50 years ago (Merrifield, 1963), continues to experience advancements in its chemistry and functional versatility (Isidro-Llobet et al., 2019). Newer experimental techniques mediated through microwave irradiation (Berrino and Supuran, 2018), photo-redox catalysis (Shaw et al., 2016), and electrochemically mediated organic synthesis (Yan et al., 2018) continue to expand the synthetic chemist's toolbox expanding the options for more versatile engineered automation solutions. Continued additions to the chemistry methodology compendium will undoubtedly lead to an expansion of the scope of chemistry that will be inherently automatable and help reduce the need and the expense for more bespoke systems developed for transformations of much narrower scope. In keeping with themes like Idea2Data (Nicolaou et al., 2019) and Design-Make-Test-Analyze (Plowright et al., 2012) artificial intelligence and machine learning or specially tailored algorithms will play ever increasing roles toward more autonomous, routine transformations that can be directly applied without human intervention to more effectively advance knowledge in the life sciences.

## Automating the Bench

One of the biggest challenges an automated synthesis lab is likely to face in a research setting is executing syntheses involving a novel pairing of reactants, reagents, and solvent. Any number of factors can impede the desired forward progress of the reaction: (a) lack of solubility of any of the reactants or reagents; (b) a mis-match in catalyst selection; (c) unknown reaction kinetics or thermodynamic distribution of products. A means of mitigating some of these issues can be accomplished by running a prescreen of the reaction conditions at a reasonable scale. Results from such a reaction screening experiments to establish key reaction conditions that need to be further optimized for reproducibility, yield and scale-up requirements. Several key factors that may influence these outcomes are addressed in this perspective.

## Retrosynthetic Analysis

Automated synthetic labs being developed now and into the future will likely be assisted if not directly interfaced with computational tools facilitate the route selection process which will likely require the development on novel workflow development interfaces coupled to reaction template databases where automated synthesis execution routines are automatically crafted and executed. Great strides have been made in retrosynthetic analysis largely driven by nearly exponential increasing computational power and refinement of algorithms that are a result of nearly half a century of slow if not steady development (Feng et al., 2018).

Many computer aided synthetic planning programs have come and gone for reasons that are not immediately clear (Szymkuc et al., 2016). In some past anecdotal recounting about such programs the perception by some users is that retrosynthesis algorithms aren't coming up with novel synthetic routes that a skilled chemist wouldn't come up with in about the same amount of time. Compounding that perception are two likely factors: (a) the inability of many algorithms to precisely adopt functional group protecting strategies that are applied holistically to a multi-step synthetic proposal; and (b) subjectively weighted scoring functions that rank order solutions from thousands encountered using somewhat arbitrarily ranked factors such as cost of raw materials, number of steps, synthetic complexity scores, etc. that lead to “unimaginative” results from the perspective of the end user. The latter factor has recently been addressed through refinements to the Synthia platform (Badowski et al., 2019).

More recently, a resurgent interest in automated synthesis and high throughput experimentation (Mennen et al., 2019) coupled with dramatic increases in computational performance and advances in synthetic technology provides a unique opportunity particularly when applied to molecules of lower complexity. The possibility that retrosynthetic algorithms can now readily identify disconnections considered trivial by the synthetic practitioner actually makes an interesting case for integrating retrosynthetic analysis with automated chemical synthesis (Nicolaou et al., 2016). It could be possible for such a system to computationally evaluate and triage hundreds if not thousands of possible synthetic targets, identifying those that can be assembled via simple automated transformations whilst also sorting out the availability of the raw materials involved and spawning such executions automatically on an appropriately configured platform.

An important deficiency to note in modern retrosynthetic algorithms is the lack of their ability to identify optimal reaction conditions or additives (e.g., catalyst-ligand combinations, solvents etc.) for executing the synthesis of a particular target. In the case of more complex targets, current algorithms also fail to identify specific protecting group strategies that are compatible with multistep sequences. Commercially available algorithms (Feng et al., 2018) often do a good job of identifying close or exact matches of transformation in the literature from references linked to the best fitting reaction rule. However, such examples may not incorporate the most effective catalyst-ligand combination or solvent that could be possibly substituted consistent with the most current state-of-the-art knowledge on the transformation.

## Reaction Templating

Having identified a potential protocol for automatic execution the next hurdle is establishing a mechanism for appropriately parameterizing its execution on an automated platform. We refer to this process as Reaction Templating. A proof of concept for this approach has recently been demonstrated in the development of the ChemPuter (Li et al., 2015). The goal of such a process needs achieve an abstraction of the general process for chemistry execution into a master schema—which may be considered a compendium of schemas of highly structured information in an extensible format (e.g., JSON, XML) that can be computationally interpreted and directly applied to machine operation and control.

**Figure 2:**
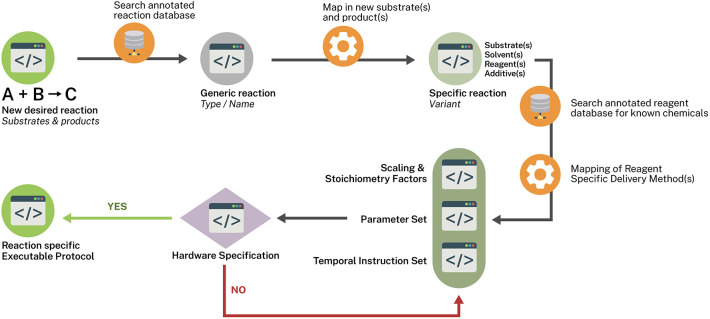
Reaction templating scheme.

A means of coupling these schemas to execution protocols may come from linking an ontologically annotated reaction database to schemas that generically represent the reaction and various key chemical participants. Such a schema (Figure 2) could then be mapped to a more specific variant of the reactant less generically represented followed by a mapping-in of novel substrates and a schema representing the parameters and scaling factors. This schema is in turn mapped to a schema of temporal instructions which in turn is mapped to hardware schema capable of executing the chemistry within the defined parameter set and instructions. A mapping exercise of the known chemicals to be involved with a reagent database annotated with reagent specific delivery descriptors (e.g., a solid-supported reagent could be delivered by a powder dispensing robot) which then map to the Temporal Instruction Set schema. Any given reaction type may in fact give rise to a variety of possible reaction vectors such as batch or flow modes. Within these modes various temporal instruction sets may exist along with stoichiometry and hardware specification considerations. A schema such as the one proposed would allow for a variety of one to many relationships to be created without sacrificing specificity or the flexibility of introducing new methodologies as long as matching hardware specifications can be successfully mapped to the implementation being queried.

## Reagent Delivery Strategies

The need to achieve the reproducibility of experimental results has be currently recognized as an important challenge across a number of scientific disciplines (Baker, 2016). One of the common features sought in the development of automated synthesis equipment is addressing the reproducibility of results. As the development of conventional platforms for liquid and solid delivery continue to advance it is likely that both the scope and precision of such equipment will improve. A recent review of a variety solid dispensing technologies surveys their strengths and weaknesses (Bahr et al., 2018).

Another means of dealing with this challenge may be to cardinalize the parameter space in such a way as to only allow delivery of pre-packaged or encapsulated units of reagent. Such pre-packaged units organized in a monetarized structure would thus allow more facile delivery of reagents and likely a much more reproducible means of precise control of variables involved in the delivery of such reagents in repeated experiments. It has recently been shown that it is possible introduce encapsulated reagents whereby reagents can be released once delivered to the target reactor under the appropriate set of conditions that release the reagent from its encapsulation (Sather et al., 2015). This form of “synthesis discretization” may permit one to rein in the exponentially high permutations that arise from a non-bounded parameter space and reduce the cost of engineered solutions. More recently a group at ETH Zurich (Jiang et al., 2019) have shown how innovative reagent design, custom hardware engineering, and a precisely executed workflow can lead to complete and repeatable workflows that lead to cleanly isolated products. In all likelihood reagent formulation will play an ever-increasing role in the development and expansion of practical automated synthetic solutions.

## Product Isolation

Product isolation refers to the process involved in separating the desired synthesis product from the reaction milieu. This milieu could include a solvent that is incompatible with downstream processing and/or byproducts resulting from spent reagents or undesired side-products involving the reactants. Product isolation often possesses the greatest challenge in providing robust automated processes. As shown in apparatus developed by scientists at ETH Zurich mentioned earlier (Jiang et al., 2019), special considerations for product isolation are likely just as important as the reaction setup itself if improved automated outcomes are desired. Confounding factors include the largely unpredictable physical characteristics of the raw reaction mixtures. Any one or combination of these characteristics may be present: (a) Solid precipitates; (b) Unexpected or unidentified byproducts; (c) changes in material viscosity (d) unconsumed reactant material requiring additional quenching.

## Purification and Characterization

More often than not, automated purification processes refer to chromatographic processes, although in process development settings, economic and scaling relegate ultimate purification efforts to re-crystallization processes. However, material constraints and lack of practical engineering solutions make the consideration of re-crystallization processes prohibitively difficult to pursue in a smaller scale laboratory setting, although there are documented methods for automated removal of solids from automated workstreams (Fitzpatrick and Ley, 2018).

Ideally, reaction isolates emerging from the product isolation process will have their overall purity significantly enhanced such that additional purification is unnecessary or sufficiently pure enough to be re-introduced for multistep synthetic applications. In the event the material does not meet purity thresholds set by business or research guidelines, it would then enter the chromatographic purification process. The process usually begins with a screening of chromatographic methods to establish the best method to achieve the appropriate recovery and purity of the material. Quite often this involves exploring the redissolution of the material in select solvents or solvent mixtures to facilitate material transfers. The lack of ability to anticipate difficulties in achieving full dissolutions presents challenges that make automating the process more difficult. Quite often manual processes are implemented to remove solids either by centrifugation or filtration then further analyses are performed to determine the makeup of these isolated solids. Given the relevance of the water solubility as key property to be optimized in the context of drug development, a great deal of effort has been spent on the *in silico* prediction (Bergstrom and Larsson, 2018). However, there is a dearth in the advancement of general solubility prediction tools in solvents other than water but is not without some recent precedent (Piccione et al., 2019).

## Role of AI/ML

The role of AI/ML will undoubtedly play a critical role in development of the lab bench of the future. Critical to the application of such techniques will require highly regularized or structured data. The proposed Reaction Template scheme (Figure 2) allows for a structured means of creating a network of discrete paths for executing chemical synthesis in a potentially highly reproducible manner. Pattern analysis of such networks by machine learning techniques might lead to the discover of new, viable, immediately executable paths. In particular, if such reaction databases can be further annotated with results of reaction screening results run within the same platform, quantitative results—both positive and negative yield results—can be used to refine the algorithm.

Further, a well-annotated reaction network has the potential to transform the way we plan synthetic routes today. That is, instead of exploring potentially untested routes to specific targets, it might be possible to identify hubs (Sudarshan et al., 2012), i.e., easily accessible intermediates or scaffolds from which the target molecules can be reached via a few number of low-risk synthetic steps. This approach can also help to reduce overall operation costs by identifying intermediates that needs to be synthesized at larger quantity in order to serve as stepping stone toward other targets. It is also possible, that new generation AI algorithms will be able to systematically reveal chemical transformations that are unknown to date (Segler and Waller, 2017). In summary, AI-based technology in chemistry not only holds the promise to facilitate the navigation of chemical transformation-space more efficiently, but it might lead to reshaping this space via the discovery of “synthetic wormholes,” i.e., novel chemistry.

## Author Contributions

AG conceived and wrote the first version of the paper. GS and SM discussed, edited the manuscript and supplied key refinements to the manuscript. GS reviewed the overall structure and content of the paper, provided critical feedback and obtained copyright permissions for all the images presented. GZ-K contributed substantially to the Role of AI/ML section.

### Conflict of Interest

The authors declare that the research was conducted in the absence of any commercial or financial relationships that could be construed as a potential conflict of interest.
